# Development of a LoRaWAN IoT Node with Ion-Selective Electrode Soil Nitrate Sensors for Precision Agriculture

**DOI:** 10.3390/s22239100

**Published:** 2022-11-23

**Authors:** Noel Bristow, Saravanan Rengaraj, David R. Chadwick, Jeff Kettle, Davey L. Jones

**Affiliations:** 1 School of Computer Science and Electronic Engineering, Bangor University, Bangor LL57 1UT, UK; 2 School of Natural Sciences, Bangor University, Bangor LL57 2UW, UK; 3 James Watt School of Engineering, University of Glasgow, Glasgow G12 8QQ, UK; 4 SoilsWest, Centre for Sustainable Farming Systems, Food Futures Institute, Murdoch University, Murdoch, WA 6105, Australia

**Keywords:** fertiliser, decision support, Internet of Things (IoT), LoRaWAN, soil nitrate, agritech, ion-selective sensor, smart agriculture

## Abstract

Crop productivity is highly dependent on the availability of soluble nitrogen (N), e.g. nitrate, in soil. When N levels are low, fertilisers are applied to replenish the soil’s reserves. Typically the timing of these applications is based on paper-based guidance and sensor-based measurements of canopy greenness, which provides an indirect measure of soil N status. However this approach often means that N fertiliser is applied inappropriately or too late, resulting in excess N being lost to the environment, or too little N to meet crop demand. To promote greater N use efficiency and improve agricultural sustainability, we developed an Internet of Things (IoT) approach for the real-time measurement of soil nitrate levels using ion-selective membrane sensors in combination with digital soil moisture probes. The node incorporates state-of-the-art IoT connectivity using a LoRaWAN transceiver. The sensing platform can transfer real-time data via a cloud-connected gateway for processing and storage. In summary, we present a validated soil sensor system for real-time monitoring of soil nitrate concentrations, which can support fertiliser management decisions, improve N use efficiency and reduce N losses to the environment.

## 1. Introduction

Nitrogen (N) is an essential nutrient for crop productivity; artificial N fertiliser has been used for decades to maintain or restore soil nutrients and to increase crop yields. This represents a large financial expenditure for farmers and therefore it is important to minimise usage. Excess N fertiliser application leads to low nitrogen use efficiency (NUE) due to N losses from volatilisation, denitrification, and surface runoff and leaching into the soil; this can cause environmental damage and human health issues [1]. These detrimental environmental issues result from our inability to accurately measure fertiliser inputs and match them to crop demand in the field. One potential approach to improve NUE is to measure the plant-available N in the soil combined with crop canopy sensors; a study by Cao et al. showed a 61–67% increase in N partial factor productivity using this approach [2]. Access to plant and soil data rapidly and inexpensively in the field remains one of the biggest challenges of precision agriculture [3,4]. Most importantly, the lack of a soil nitrate (NO_3_^−^) measurement system is a major impediment to improving precision agriculture technology; the development of real-time or near real-time measurement systems could facilitate more appropriate decision-making in the agriculture industry [5]. Although the majority of studies have utilised laboratory and field scale soil NO_3_^−^ testing of soil samples and not real-time sensing in-situ, one study in North America by Zhu et al. has used solid-state NO_3_^−^ sensing for sixty days in a field setting with wired sensors [6,7,8,9]. None of these have reported validated NO_3_^−^ sensors, assessing N sensor functionality for improving N-fertilisation, assimilation, and transformation in agricultural soil. Monitoring tools capable of providing online, in-situ, continuous temporal soil NO_3_^−^ measurements are crucial for an agri-tech revolution that aims to use precision technology. Although a range of sensing technologies have been proposed for monitoring soil N status, few have been used outside the laboratory [10]. This is due to the problems of cross-reactivity with other ions in solution, poor resolution, sensor drift, and lack of robustness in response to environmental changes that can occur in the field (e.g. drought, flooding, freezing, mechanical damage).

Here we describe an ion-selective electrode (ISE) approach used for measuring NO_3_^−^ concentrations in soil, similar to those used for freshwater [11,12]. We focused on NO_3_^−^ as it is a commonly used form of N fertiliser and is the dominant form of N taken up by most crop plants from agricultural soils [11]. In addition, NO_3_^−^ does not sorb to the soil’s solid phase, is highly mobile in soil and thus the concentration in soil solution provides a reliable estimate of the available N stocks held in soil [12].

The requirement in precision agriculture for real-time remote sensing can be met by taking an Internet of Things (IoT) approach. The LoRaWAN wireless protocol is low power, long range, and low data rate and therefore is an ideal technology for field sensor datalogging; the data rate required for measuring soil conditions is low, dataloggers are usually battery powered, the network infrastructure is relatively easy to install, and the nature of agricultural sensing (collecting data from widely distributed dataloggers in the outdoors) hampers manual data collection [13,14,15]. Although a few LoRaWAN agriculture nodes have been developed, some including SDI-12 soil moisture probes, this is the first time that soil NO_3_^−^ sensing has been incorporated into an agricultural IoT node [16].

The challenge addressed by this paper is to deploy NO_3_^−^ sensors in a field context, having addressed the issues of sensor lifetime and validation. In addition, the sensors are connected to a LoRaWAN-enabled datalogger, allowing remote sensing of soil NO_3_^−^ levels, alongside soil moisture measurements. This will allow farmers to monitor field N levels from the comfort of their offices, or on their phones. This is the first time remote field-scale sensing of NO_3_^−^ has been possible and work of this nature has not been published before. The system described here comprises a battery-powered datalogger, supporting four SDI-12 soil moisture sensors and eight bespoke soil NO_3_^−^ sensors, connected to the internet wirelessly via LoRaWAN, allowing measurements to be taken every fifteen minutes and transmitted in real-time to a cloud server for instant remote access.

## 2. Nitrate Sensor Development

For more than two decades, researchers have attempted to develop a real-time soil NO_3_^−^ measurement system: ion-selective field effect transistor; ion-selective electrode; spectrophotometer; lab-on-a chip technologies. These studies have not progressed beyond the laboratory and testing in soil columns. A recent review, however, shows that some of the barriers to implementing a field-based real-time soil NO_3_^−^ measurement system have been overcome [17]. The review describes various methodologies and procedures for data acquisition to measure and analyse soil NO_3_^−^ levels with the existing sensor technology. In particular, advances in the in-field use of NO_3_^−^ ISE systems seem promising. These provide the means for in-field monitoring of soil NO_3_^−^, assessing spatial and temporal variability of soil NO_3_^−^ concentrations, and developing site-specific fertiliser applications (i.e. precision agriculture).

### 2.1. Ion-Selective Electrode Soil Nitrate Sensors

**Figure 1:**
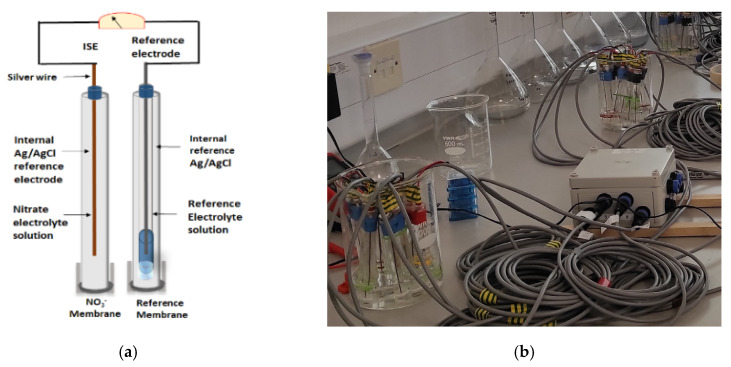
Ion-selective electrodes for nitrate sensing: (**a**) schematic representation of an ion-selective electrode; (**b**) prototype nitrate sensors connected to an IoT sensor node.

Ion-selective electrodes represent the predominant method for measuring NO_3_^−^ concentrations outside of the laboratory (Figure 1a). The ISE concept was developed during the 1970s by incorporating ion-exchange salt and an ionophore within plasticised flexible polymer membranes [18].

Such electrodes have been predominantly deployed for the monitoring of freshwater rather than soils [19]. ISEs show good accuracy and have fast response times with limits of detection (LOD) in the range 0.1 to 0.7 μM, making them suitable for rapid screening of NO_3_^−^ concentration in soil extracts [20,21,22]. This concept has been extended to on-the-move rapid soil NO_3_^−^ testing, where the NO_3_^−^ ISEs are mounted on a vehicle (e.g. tractor-mounted) that is coupled to automated soil samplers and extraction platforms [23,24,25,26]. Typically, this on-the-move system continuously takes soil samples at 0–15 cm depths as the vehicle moves through the field. Whilst on-the-move sampling represents an improvement from on-farm manual rapid-tests, each sampling event entails an economic cost and significant changes in soil N status may occur between infrequent sampling events. Essentially, the extent to which farmers will adopt on-the-move testing and manual rapid-tests is not clear, and in general, there is a lack of information in the literature detailing how, when, and if farmers will adopt these on-the-move methods within farming operations. Hence, the development of sensors capable of in-situ real-time determination of soil NO_3_^−^ may represent a preferred solution for precision agriculture. Sensor networks deployed in the field during the growing season may enable continual monitoring of soil N, allowing a dynamic approach to fertiliser management that responds quickly to changing agronomic conditions [17].

### 2.2. Overview of the Nitrate Soil Sensor Approach

Here we present a simple, low-cost, field-embeddable soil N sensor capable of detecting real-time soil NO_3_^−^ concentration. This method is a membrane-based ion-selective measurement of NO_3_^−^ concentration in the soil pore water and the analytical procedures enable continuous in-situ monitoring of soil NO_3_^−^ over the growing season. Additional measurements of the soil moisture content are required to calculate the NO_3_^−^ stock available for plant uptake. Hence, Acclima TDT-SDI-12 soil moisture and temperature sensors were placed next to the NO_3_^−^ sensors in the field. The field validation studies were conducted for 3–4 month periods during forage maize (2018) cultivation. The promising laboratory and field data show the potential of these improved soil sensors for in-situ, real-time soil NO_3_^−^ detection. The field validation was accomplished using several sensors connected to an IoT node, the measurements being physically retrieved on an ad-hoc basis. The subsequent challenge is to enable a wireless sensor network system for data collection, where the data would feed directly into models to generate fertiliser recommendations. We believe that the proposed monitoring technology could open a new avenue for precision fertilisation and optimisation of crop production while reducing the risks associated with environmental pollution.

### 2.3. Manufacture of the Nitrate Sensors and Reference Electrodes

To make the NO_3_^−^ sensors, a mixture of NO_3_^−^ ionophore (tridodecylmethylammonium nitrate, 6 wt. %), PVC (23 wt. %), nitrocellulose (5 wt. %), and 2-nitrophenyl octyl ether (66 wt. %) were dissolved in tetrahydrofuran. PTFE membranes (5 μm pore size, 125 μm thickness, 13 mm diameter) were then coated with the ionophore solution and the membranes and allowed to dry for 24 h. The reference electrode was made in a similar way but contained potassium tetrakis (4-chlorophenyl) borate (1 wt. %) as an ion-exchanger, alongside polyethylene glycol 1500 (47 wt. %), PVC (47 wt. %), and nitrocellulose (5 wt. %). The dry NO_3_^−^ and reference membranes were then embedded in separate 15 cm long poly(methyl methacrylate) (PMMA) electrode bodies (RS Components Ltd., Corby, UK), sealed with rubber O-rings, and further sealed with silicon sealant (UniBond). This produced a 10 mm diameter flat PTFE-based sensing membrane [27]. The reference electrode is an Ag/AgCl type with a double junction that consisted of an outer reference membrane sleeve and inner glass frits liquid junction-based electrode. An Ag wire was electroplated with the chloride (3 M KCl solution) and inserted into the inner tube of the double-junction electrode. Glass frits with approximate dimensions of 3 mm diameter, 3 mm length, and 4 nm pore size were attached to the inner tube of the double-junction electrode using heat shrink tubing. The inner filling solution contained 50 mM of KCl while KNO_3_ was added to the NO_3_^−^ electrode. Both the inner and outer of the double junction reference electrode were filled with 3 M KCl (saturated with AgCl). The N-sensor terminal leads from both electrodes were connected to the data logger using an external wire.

### 2.4. Nitrate Analysis Technique

The electrodes were conditioned with 100 mM of NO_3_^−^ solution overnight to equilibrate the membrane until a stable voltage was obtained. The voltage was continuously monitored at room temperature (22 ± 1 °C) under non-stirring conditions. After conditioning, electrodes were calibrated with six NO_3_^−^ standards with different concentrations of NaNO_3_ (from 10^−6^ M to 10^−1^ M). The ion-selective electrode generates a voltage across its membrane that varies with the molarity of the solution. The measured potential difference is proportional to the logarithm of NO_3_^−^ ion concentration. Electrode potential was calculated using an experimentally determined Nernstian slope response against the logarithmic NO_3_^−^ solution concentration [28]:

*E* = *E*^0^ − (*RT*/*nF*) log *C* (1)

where *E* is the electrode potential (in volts), *E*^0^ is the standard reduction potential (in volts), *R* is the gas constant (8.314 J·K^−1^·mol^−1^), *T* is the temperature (Kelvin), *n* is the charge of the ion, *F* is the Faraday constant (96,500 coulombs·mol^−1^), and *C* is the reaction quotient that represents activities or molar concentrations.

Normally N sensors are calibrated with known standards to obtain the calibration curve. The voltage output is related to logarithm of solution molarity, which is used to determine the NO_3_^−^ concentration of subsequent soil pore water measurements. The resulting voltage from the electrode is then mathematically converted to soil NO_3_^−^ concentration using the calibration curve. Environmental variables such as moisture and temperature were considered when the electrode voltage was processed to calculate the actual soil NO_3_^−^ content. N sensors were periodically removed from the field and tested for re-calibration checks to monitor the calibration shift in the slopes.

### 2.5. Methodology Used to Calibrate the Sensors

**Figure 2:**
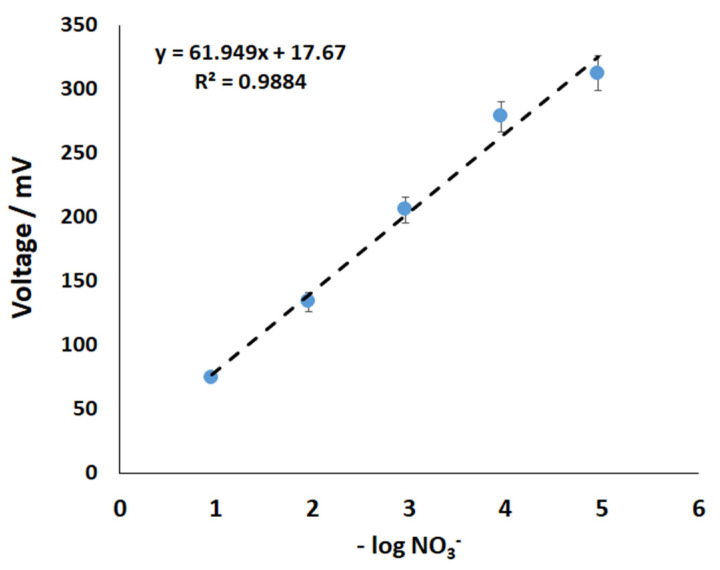
Nitrate sensor voltage response vs. nitrate (NaNO_3_) solution concentration (calibration graph shown here is the negative log of molar concentrations). (See Table S1).

Figure 1b shows the prototype N sensor connected to the logger capable of continuous in-situ measurement of NO_3_^−^ concentration in the soil pore water. The N sensor response time is one of the most critical factors in developing a real-time sensing system. To investigate the response characteristics, N sensors were tested with different NO_3_^−^ solutions (from 10^−5^ M to 10^−1^ M). When exposed to different NO_3_^−^ solutions, the response obtained with the N sensors was fast and reproducible. Figure 2 shows the calibration line of the NO_3_^−^ sensors (n = 12). All the sensors exhibited a linear response within the range of 10^−5^ M to 10^−1^ M (detection limit of ~10 µM) with the Nernstian slope of ~62 mV/decade (R^2^ = 0.988) when tested under the non-stirring conditions at room temperature 23 ± 1 °C.

**Figure 3:**
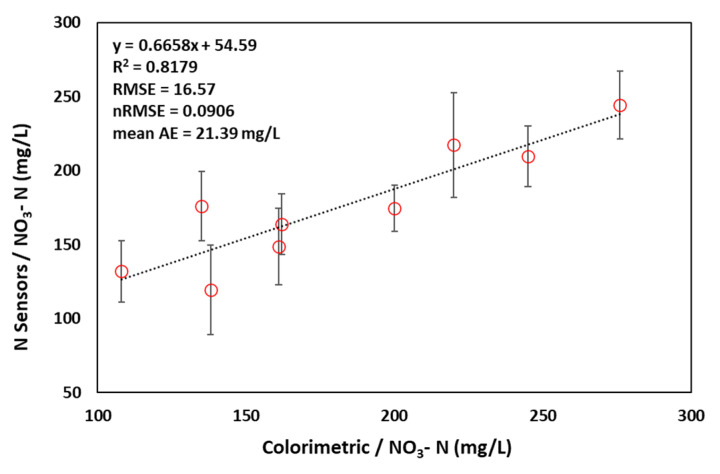
Correlation and regression between the nitrate levels measured by the soil N sensor and laboratory-based soil extraction and colorimetric analysis over a 3-month period. (See Table S2).

Figure 3 shows the correlation of N sensor readings compared with the laboratory-based soil NO_3_^−^ analysis of soil collected near the sensors (0.5 M K_2_SO_4_ soil extracts and colorimetric analysis). Overall the data compared favourably with a correlation of R^2^ = 0.82 between the two techniques. The difference found between the two techniques is most probably due to the high spatial variability in soil NO_3_^−^ concentrations and the production of NO_3_^−^ that may occur following laboratory handling of soils and during the extraction process [29,30]. At present this N sensor shows great potential to be used for in-situ soil NO_3_^−^ measurements, however, further refinements are in progress to extend the sensor lifetime for longer operation in soil (i.e. >6 months).

After calibration and stability checks, sensor performance was validated in a temperature-controlled laboratory using topsoil samples from a pasture field in Abergwyngregyn (North Wales, UK) in a pot experiment. The soil was a Eutric Cambisol and had a clay loam texture. There were two sets of treatments: (i) one to measure the addition of NO_3_^−^ to the soil (in a solution containing 210 mg·L^−1^ N) and (ii) the same volume of deionised water as the NO_3_^−^ treatment (i.e. the control). There were six replicates of each treatment, with one NO_3_^−^ sensor per pot/replicate. The sensors were able to detect the presence of NO_3_^−^ in the topsoil (10 cm), with a clear difference in the signal observed between the control and with NO_3_^−^ addition (data not shown). Such an N sensor response in lab-based soil testing is promising for the deployment of sensors for field testing.

### 2.6. Field Testing

**Figure 4:**
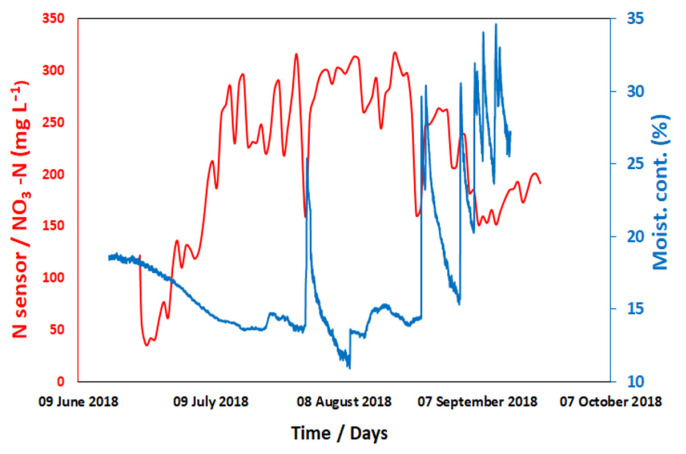
Soil nitrate concentration (at 10 cm depth) was measured over a period of three months by N sensors plotted alongside soil moisture data from soil moisture sensor (Acclima TDT-SDI-12 soil moisture sensor). (See Table S3).

Field trials were conducted during the 2018 growing season at the Henfaes Research Centre, Abergwyngregyn, Wales (53°14′19″ N, 04°00′55″ W). NO_3_^−^ sensors were connected to GP2 dataloggers (Delta-T-Devices, Cambridge, UK) which support the 12 analogue inputs with the readings combined into a single dataset. The output from the N sensor successfully tracked NO_3_^−^ in the soil during the whole field trial. Figure 4 shows NO_3_^−^ concentrations measured using the soil sensors over a period of three months plotted alongside soil moisture data (Acclima TDT-SDI-12 Soil Moisture Sensor). Overall, this shows soil NO_3_^−^ concentrations increased during the early growing season in response to a progressive reduction in soil water content (which concentrates NO_3_^−^) and the rapid nitrification and production of NO_3_^−^ which occurs in this soil [31]. It was observed that the N sensor’s reading was responsive to changes in soil moisture content. The soil moisture curve shows a rapid and strong response to individual rainfall events (e.g. in early August), leading to a short-term reduction in the N sensor reading due to a dilution of NO_3_^−^ in the soil water. In September repeated rainfall events lead to a longer-lasting increase in soil moisture content and a reduction in soil NO_3_^−^ levels (due to dilution). Even in extreme drought conditions, the sensors continued to give readings, though some discrepancy in data is expected during dry soil conditions.

The sensor output voltages were converted to NO_3_^−^ concentration (in N mg·L^−1^) based on their individual calibration values (Nernstian slope). By employing soil temperature and moisture compensation using Equation (1), the resulting NO_3_^−^–N mg/L concentration was subsequently converted to kg N ha^−1^ by using Equation (2).

N-sensor kg N ha^−1^ = (Value in NO_3_^−^–N mg·L^−1^) × (1 kg/10^6^ mg) × (1000 kg/m^3^, bulk density) × (0.1 m × 10^4^ m^2^·ha^−1^) (2)

To convert between N sensors real-time data of soil NO_3_^−^–N mg·L^−1^ to kilograms per hectare (kg·ha^−1^) requires values for the soil bulk density (kg·m^−3^) and the depth of N Sensor placement (metres). Assuming soil bulk density is 1000 kg·m^−3^ per hectare the final readings kg N ha^−1^ can be compared to the fertiliser application rate.

## 3. Wireless Networking of Soil Sensors

Having proved the efficacy of these NO_3_^−^ sensors during the 2018 growing season the testing program was extended to other field trials. Frequent data retrieval from diverse sites would be problematic so integration with an Internet of Things (IoT) wireless node enables measurements to be made at remote sites and the data collated and processed on a central server without a manual collection of the data. Initially, the system has been designed so that each node can support eight soil NO_3_^−^ sensors, as well as up to four soil moisture sensors (Acclima TDT-SDI-12 soil moisture sensor), with measurements taken every fifteen minutes.

### 3.1. IoT Soil Sensor Node Design

**Figure 5:**
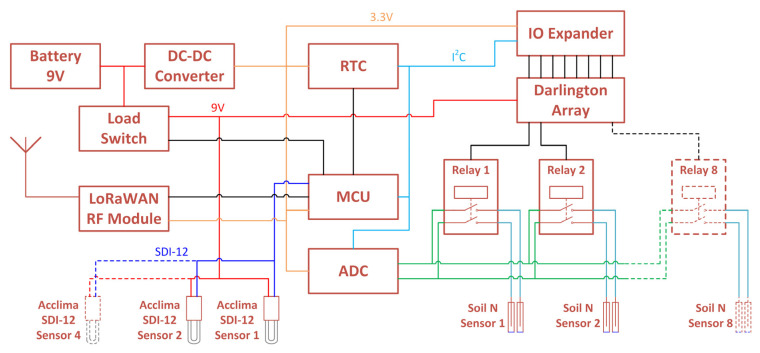
Design overview of IoT soil sensor node.

Figure 5 shows an overview of the IoT soil sensor node. The node was originally designed around the ATmega328P microcontroller (MCU), as this allowed rapid prototyping of both the hardware and software. Once radio communications were added, the MCU was upgraded to the ATmega644PA, as this has multiple hardware serial ports and greater on-board memory.

The node comprises the following principal components:

- ATmega644PA—microcontroller (MCU)
- DS3231M—precision real-time clock (RTC)
- MCP23017—16-channel I^2^C I/O expander
- ULN2803—8-channel Darlington transistor array
- TPS27081—high-side load switch
- TPS62745—ultra-low I_Q_ step-down DC-DC converter
- ADS1115—16-bit analog-to-digital converter (ADC)
- RN2483A—LoRaWAN radio module

Sensor inputs:

- 8× soil NO_3_^−^ ISE sensors—multiplexed by relays
- 4× Acclima TDT soil moisture sensors—SDI-12 bus

Each of the soil N sensors is multiplexed to the ADC using relays selected by the I/O expander and Darlington transistor array. The Acclima soil moisture sensors are all connected to a single SDI-12 serial bus, with each sensor having a unique address pre-programmed before being connected to the node. The Acclima sensors measure four soil parameters: volumetric water content, soil temperature, bulk relative permittivity, and soil electric conductivity.

**Figure 6:**
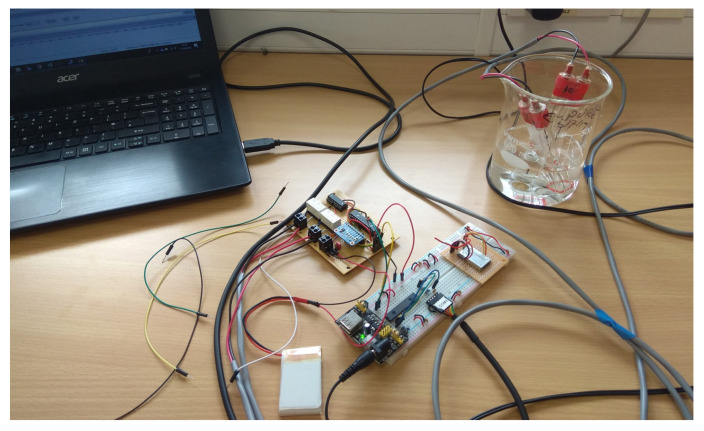
Prototype IoT soil sensor node undergoing laboratory tests.

Figure 6 shows an early prototype sensor node undergoing laboratory tests, with two N sensors multiplexed to the node and immersed in a solution of known NO_3_^−^ concentration. The aim was to check that the multiplexing and relays were working correctly (i.e. they were able to switch between the N-sensors) and that the ADC was able to correctly measure the output voltage from the N sensor as this has a high impedance output. The voltages measured by the prototype IoT node were within 0.3% and 0.2% respectively of the voltages as measured by a Delta-T GP2 datalogger. 

### 3.2. Wireless Communications

The communication requirements for these nodes are for a small amount of data to be sent at fifteen-minute intervals from field locations. Such time intervals enable data collection during extreme events to be collected (e.g. torrential rain) as well as long-term monitoring. There are several technologies and protocols available for wireless low-power, wide-area networks, but in this case, LoRaWAN is ideal as it has a low data rate, small packet size, and long range. LoRaWAN is a network stack protocol operating on the LoRa physical layer, operating at 868 MHz in Europe [32]. It is low power and has a long-range (2–10 km), utilising public gateways to communicate with the internet [33,34]. In this node, a Microchip RN2483A radio module has been used as it has the full LoRaWAN stack built into the module which reduces the coding requirements of the MCU [33]. The RN2483A can be put into sleep mode which keeps the power requirements down.

**Figure 7:**
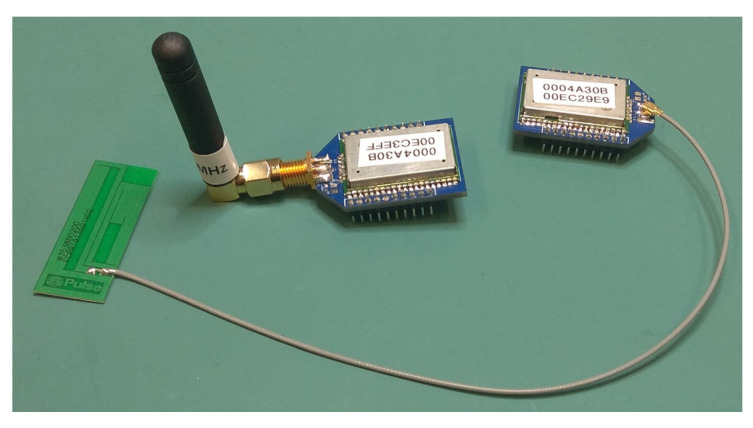
LoRaWAN RN2483A communication modules: one with an adhesive PCB antenna and the other with a stub helical coil.

A separate PCB was designed for the RN2483A RF module so that each module could be pre-configured on an external circuit before being plugged into the soil sensor node. The PCB was designed so that it could be fabricated with either a U.FL or an SMA RF socket, allowing a choice of aerials to be attached (Figure 7).

The Things Network (TTN) is an open-source, free-of-charge network infrastructure based on the LoRaWAN protocol. There is an ever-growing network of TTN gateways that will route incoming LoRaWAN data packets to the TTN network servers. LoRaWAN is a secure protocol and each module must be programmed with its own security keys [35]. For our work, a separate circuit was built to allow each RN2483A module to be pre-configured with its unique keys using Over The Air Activation (OTAA). At the same time, the module is also configured to use European radio frequencies and settings. These settings are all stored in RN2483A’s EEPROM, so it can be plugged into a sensor wireless node and used without any more intervention.

### 3.3. Power Requirements

Although wireless sensors such as these are often powered by solar energy harvesting, in this case, it was decided that battery power would supplement the performance as they were only going to be deployed for a few months at a time during the growing season and would be in an environment subject to shading from growing plants during the later months. The Acclima soil sensors require at least six volts, so a battery pack of 6xAA batteries was used. Apart from the SDI-12 bus and the relays, the rest of the node runs at 3.3 V (obtained by the TPS62745 DC-DC converter). To minimise power losses, the battery is only connected to the relays and SDI-12 circuitry when the node is taking measurements and otherwise is disconnected; this is controlled by the TPS27081 load switch. The MCU is put into deep sleep mode between measurements, being woken up at the required time by the RTC polling an interrupt pin on the MCU. The RN2483A is also put into sleep mode between transmissions.

## 4. Wireless Node Operation

**Figure 8:**
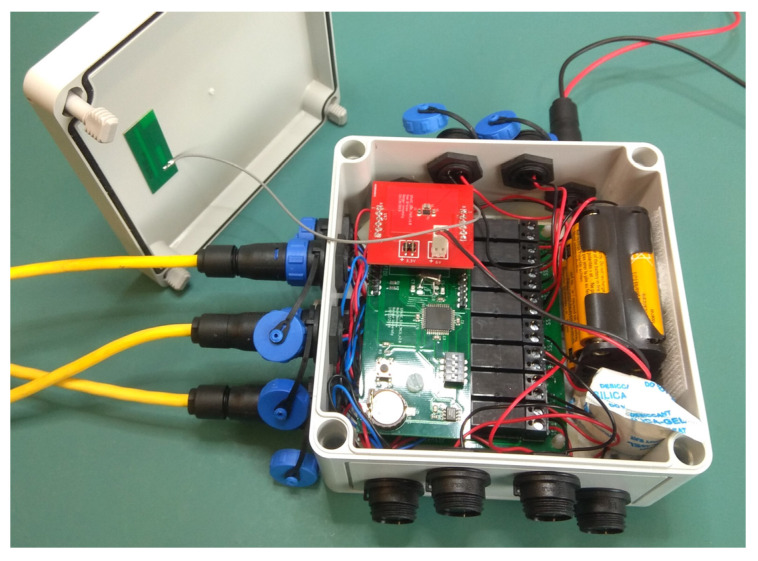
Complete IoT soil sensor node ready for testing.

Figure 8 shows the completed IoT soil sensor node ready for testing. It has been built on several interconnected PCBs to allow for further modification as required.

### 4.1. Node Initialisation

When the node is powered up, it will first command the RN2483 RF module to attempt to join the network, giving it time to initialise. Next, the node will identify which N sensors are connected (selected by DIP switches) before querying the SDI-12 bus for Acclima soil moisture sensors (each having been pre-programmed with unique addresses).

### 4.2. Measurement Cycle

When the node is due to take its next set of measurements the RTC will poll the interrupt pin on the MCU to wake it up. The MCU will then power up the relays and SDI-12 circuitry, giving the Acclima sensors time to power up, and wake up the RN2483 LoRaWAN module. The MCU will process through each soil N sensor: switching on its relay to connect it to the ADC, reading the voltage from the ADC (after a short settling time), switching off its relay. Once the MCU has read all the soil N sensors it will send out a single group command to all connected SDI-12 sensors to take a measurement. The MCU will then retrieve the measured data from each SDI-12 sensor before powering down the relay and SDI-12 circuitry. Having taken all the measurements the MCU will build the payload message with the results, transmit it over LoRaWAN and then put the LoRaWAN module to sleep. Finally, the MCU will program the RTC with the next wake-up time and put itself to sleep, ready for the next measurement cycle.

## 5. Data Processing

**Figure 9:**
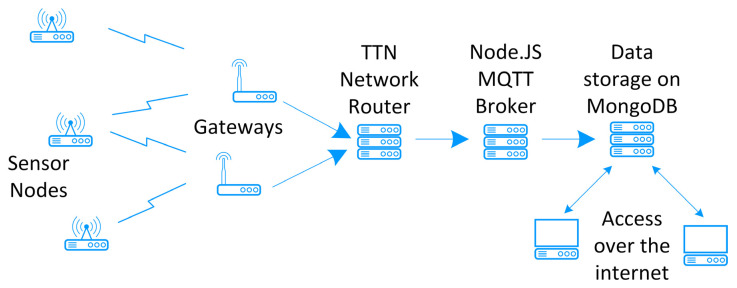
Overview of data processing.

The gateways receive the data packets from the sensor nodes and forward them to the TTN network router. The TTN router is programmed to forward incoming data traffic to a Node.js MQTT (Message Queuing Telemetry Transport) broker, where the data can be extracted from the message and stored on an online database (MongoDB). Once the data has been stored, it can be queried and analysed as required over the internet (Figure 9).

## 6. Deployment

**Figure 10:**
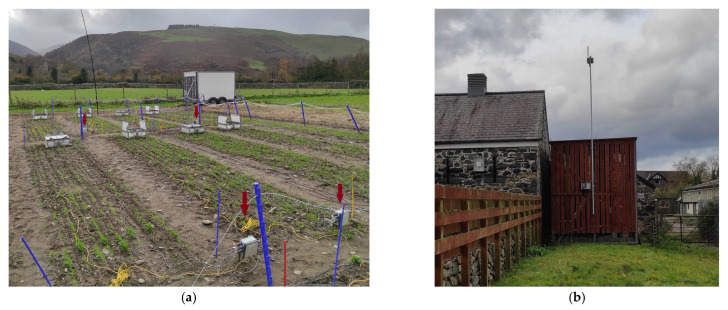
Deployment at Henfaes Research Farm, Abergwyngregyn: (**a**) nodes (arrowed) deployed in a wheat crop (the large, lidded boxes are measuring greenhouse gases); (**b**) MultiTech LoRaWAN gateway.

Seven nodes were deployed at Henfaes, Bangor University’s Research Farm at Abergwyngregyn, over the 2021 growing season to monitor soil NO_3_^−^ concentrations under a winter wheat (*Triticum aestivum* L.) crop (Figure 10a) [36]. Due to its remote location, it was necessary to install a LoRaWAN gateway (MultiTech External IP67 gateway, MTCDTIP-266A-868) at Henfaes (Figure 10b) [37]. Henfaes has an automated weather station with a telemetry data transfer (Campbell Scientific), which was used for rainfall and meteorological data.

## 7. Results

**Figure 11:**
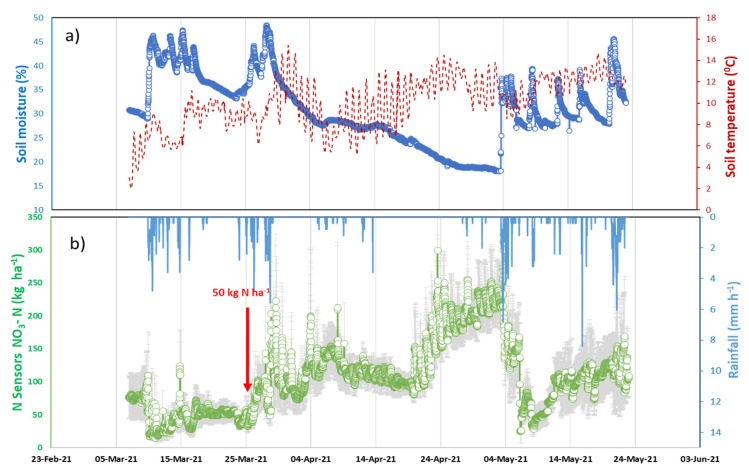
Sample results from Henfaes deployment: (**a**) soil sensor temperature and moisture; (**b**) soil sensors nitrate measurements with fertiliser applications highlighted, plotted against the rainfall. (See Table S4).

Figure 11 shows the results from the field testing of the arrays of N sensors and soil moisture and temperature sensors connected to the LoRaWAN IoT nodes. All sensors were calibrated before the field deployment during the wheat field trial. Figure 11a shows the plot of soil temperature and moisture sensor trends. The rain-fed wheat field trial resulted in soil moisture ranging between 20–45% with the lowest soil moisture content of 20% recorded at the end of April. Soil sensors showed a low soil temperature of 2 °C at the beginning of the trial which then gradually increased. In addition, sensors measured the diurnal soil temperature variations observed during the trial.

Rainfall (data collected from the nearby weather station) was distributed throughout the wheat-growing season, with intensive rainfall ranging from 4–8 mm·h^−1^ occurring between March and May (Figure 11b). The soil moisture content trends were relative to rainfall events as the soil sensors showed a proportional increase in soil moisture. Similarly, the soil NO_3_^−^ sensors responded to the rainfall-driven NO_3_^−^ soil movement, which corresponds to the spikes in the signal (Figure 11b). The NO_3_^−^ sensors showed a clear signal difference before and after fertilisation, which is indicated by the arrow. The sensor readings before the fertiliser application were ca. 50 kg N ha^−1^ which then increased to average readings of ca. 100 kg N ha^−1^ after the addition of 50 kg N ha^−1^ N fertiliser (in the form of ammonium nitrate). At the end of April, the measurement by the NO_3_^−^ sensor reached 200 kg N ha^−1^ before it decreased gradually with rainfall events. The signal increase could be due to the existence of low soil moisture content with prolonged soil dryness in the absence of rain and also due to the localised nitrification process in the soil. A large decrease in NO_3_^−^ sensor signals was observed during the beginning of May, indicating both plant uptake of NO_3_^−^, and also the potential downward movement of NO_3_^−^ through the soil to below the soil sensors as a result of cumulative rainfall flow through the soil.

## 8. Discussion

Here we have demonstrated the development and deployment of soil NO_3_^−^ sensors alongside off-the-shelf soil moisture and temperature sensors connected to LoRaWAN IoT nodes which will enable farmers to monitor nutrient availability. The real-time soil NO_3_^−^ concentration, soil moisture content, and soil temperature data reflected the environmental and management conditions for 3 months, with the data being successfully transmitted wirelessly via a new LoRaWAN logger. Such systems have the potential to provide real-time decision support for farmers in optimising the use of N inputs (fertilisers and organic manures), which is crucial for avoiding over or under-application of N to improve NUE and sustainable crop yields. These monitoring devices were installed in the field, which was in operation for six months providing continuous data transfer from the field, although the three months of data presented here are relevant to the observed changes with the fertiliser application and seasonal soil condition variations. N sensor development is one of the key research areas where such soil deployable sensors can be significant in real-time in-situ soil NO_3_^−^ monitoring to improve NUE.

**Figure 12:**
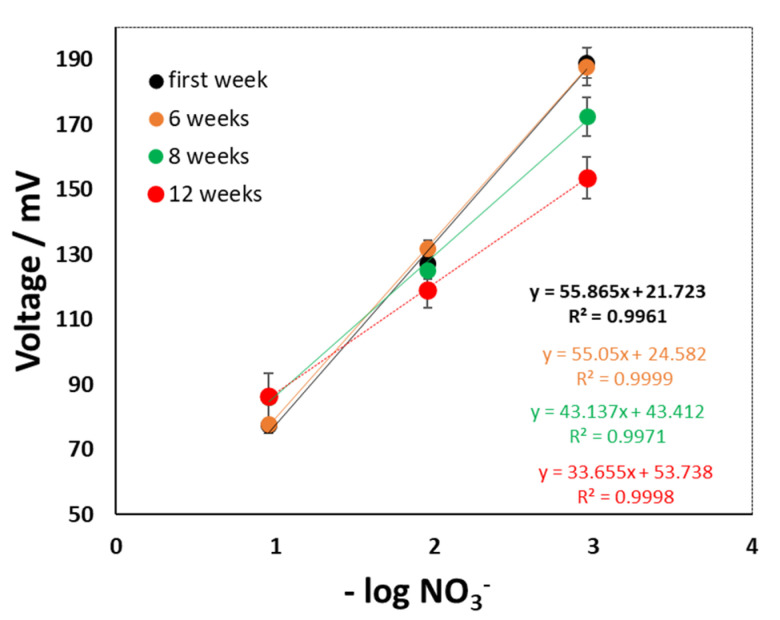
Calibration potential drift profile during the field trial.

Although the sensors showed good response sensing soil NO_3_^−^ levels, sensor signal drifts were evident during the field trial. The sensors were checked for re-calibration after 6, 8, and 12 weeks following deployment (see Figure 12). The sensors were stable for the first six weeks of operation and thereafter showed drifts in signal from the 8th week onwards. When tested on the 12th week, the sensor calibration slope deviated from the initial 55 mV to 33 mV. Therefore the development of an algorithm is essential to compensate for NO_3_^−^ sensors calibration drift to correct signals to read the actual NO_3_^−^ concentration from the field measurements.

During their deployment, two IoT nodes stopped working and were replaced by spares. It was found that the ADC was unable to successfully measure the sensor voltage during periods of drought when soil moisture levels were very low. After the investigation, it was deduced that the ADC differential input impedance was too low (4.9 MΩ at full-scale resolution (FSR) of ±2.048 V). By changing the FSR to ±4.096 V the differential input impedance was increased to 15 MΩ and this was found to be sufficient to read the sensor voltage even during periods of drought. This did not have any detrimental effect on the sensor readings, as the ADC used had a resolution of 16-bit, nominally giving voltage measurements to the nearest ±0.1 mV at the higher FSR. All the IoT nodes were reprogrammed with the new version of the firmware and re-deployed successfully. No problems were found with the SDI-12 soil sensor measurements throughout the deployment. It was found necessary to replace the batteries every 2–3 months (more often during the colder winter months). Apart from this, the nodes worked as expected and produced excellent results. The benefits of not having to make frequent trips to collect data manually (whatever the weather) and being able to continually monitor field conditions remotely is not to be underestimated.

The IoT nodes transmitted the NO_3_^−^ sensor reading as a voltage which after download from the cloud was subsequently converted to N mg·L^−1^ and finally to kg N ha^−1^. It should be possible to load each sensor’s calibration value (Nernstian slope) onto the node and perform the conversion calculations onboard, outputting a final measurement in the units required (e.g. kg N ha^−1^). This would require stable N sensors which could maintain a constant calibration value across the whole deployment, or have a known drift pattern. This would give the advantage of not needing any post-processing to obtain the results in the desired units.

## 9. Conclusions

This paper has investigated the development of an IoT soil sensor node. An ion-selective electrode sensor has been developed to detect NO_3_^−^ levels in the soil. An IoT wireless node has been designed to support eight soil N sensors and four soil moisture sensors. LoRaWAN and TTN have been selected for data transmission and cloud processing, due to their ease of use and data processing capability. The IoT soil sensor node can be readily deployed for real-time monitoring and analysis of soil conditions. Additional research is now needed to determine how to use the data generated from the sensors to provide specific farmer guidance, based on the measured soil NO_3_^−^ stock and the N response of a crop at any given period during the growing season. Overall, these results show the capability of LoRaWAN IoT nodes for remote field measurements, allowing remote access to in-situ field data in real time.

## Data Availability

The data presented in this study are available in supplementary material.

## Supplementary Materials

The following supporting information can be downloaded at: https://www.mdpi.com/article/10.3390/s22239100/s1.
